# Linking preterm infant gut microbiota to nasograstric enteral feeding tubes: exploring potential interactions and microbial strain transmission

**DOI:** 10.3389/fped.2024.1397398

**Published:** 2024-06-17

**Authors:** J. Jara, C. Alba, R. Del Campo, L. Fernández, M. Sáenz de Pipaón, J. M. Rodríguez, B. Orgaz

**Affiliations:** ^1^ Department of Galenic Pharmacy and Food Science, School of Veterinary Sciences, University Complutense of Madrid (UCM), Madrid, Spain; ^2^ Department of Nutrition and Food Science, School of Veterinary Sciences, University Complutense of Madrid (UCM), Madrid, Spain; ^3^ Department of Microbiology, Ramón y Cajal University Hospital and Ramón y Cajal Health Research Institute (IRYCIS), Madrid, Spain; ^4^ Department of Neonatology, La Paz University Hospital of Madrid, Madrid, Spain; ^5^ Department of Pediatrics, Autonoma University of Madrid, Madrid, Spain

**Keywords:** preterm infants, gut microbiota, nasogastric enteral feeding devices, hospital acquired infections, pathobionts, biofilms, late-onset sepsis

## Abstract

**Introduction:**

Preterm birth is a growing problem worldwide. Staying at a neonatal intensive care unit (NICU) after birth is critical for the survival of preterm infants whose feeding often requires the use of nasogastric enteral feeding tubes (NEFT). These can be colonized by hospital-associated pathobionts that can access the gut of the preterm infants through this route. Since the gut microbiota is the most impactful factor on maturation of the immune system, any disturbance in this may condition their health. Therefore, the aim of this study is to assess the impact of NEFT-associated microbial communities on the establishment of the gut microbiota in preterm infants.

**Material and methods:**

A metataxonomic analysis of fecal and NEFT-related samples obtained during the first 2 weeks of life of preterm infants was performed. The potential sharing of strains isolated from the same set of samples of bacterial species involved in NICU's outbreaks, was assessed by Random Amplification of Polymorphic DNA (RAPD) genotyping.

**Results:**

In the samples taken 48 h after birth (NEFT-1 and Me/F1), *Staphylococcus* spp. was the most abundant genera (62% and 14%, respectively) and it was latter displaced to 5.5% and 0.45%, respectively by *Enterobacteriaceae*. Significant differences in beta diversity were detected in NEFT and fecal samples taken at day 17 after birth (NEFT-3 and F3) (*p* = 0.003 and *p* = 0.024, respectively). Significant positive correlations were found between the most relevant genera detected in NEFT-3 and F3. 28% of the patients shared at least one RAPD-PCR profile in fecal and NEFT samples and 11% of the total profiles were found at least once simultaneously in NEFT and fecal samples from the same patient.

**Conclusion:**

The results indicate a parallel bacterial colonization of the gut of preterm neonates and the NEFTs used for feeding, potentially involving strain sharing between these niches. Moreover, the same bacterial RAPD profiles were found in neonates hospitalized in different boxes, suggesting a microbial transference within the NICU environment. This study may assist clinical staff in implementing best practices to mitigate the spread of pathogens that could threaten the health of preterm infants.

## 1 Introduction

Preterm birth, which includes neonatal births under 37 weeks of gestation, is a growing problem occurring in 5%–13% of all birth cases due to different factors, such as lifestyle, toxic habits, pregnancies at older ages or multiple pregnancies associated to *in vitro* fertilization (1). Most preterm neonates stay hospitalized in Neonatal Intensive Care Units (NICUs), an environment characterized by the abundance of nosocomial pathogens (2, 3). This fact, together with a delayed contact with the mother and family and some medical practices, including the widespread use of antibiotherapy, usually leads to an alteration in the acquisition and composition of the gut microbiota, which is frequently described as aberrant in comparison with that of full-term infants (4, 5). In addition, such combination of factors favors the selection of high risk clones and, therefore, may predispose to the acquisition of hospital-related infections (6–8).

Other factors that have an impact on the establishment and evolution of the gut microbiota of preterm neonates include the type of delivery (cesarean section, vaginal delivery) and feeding (own mother's milk [OMM], donor human milk [DHM], infant formula, mixed feedings), including the way in which feeding is provided (9–11). In this sense, feeding of preterm infants often require the use of nasogastric enteral feeding tubes (NEFT) because of their frequent inability to develop a proper suck-swallowing reflex (12, 13). NEFTs can be easily colonized by hospital-associated pathobionts. Additionally,

Additionally, the process of aspirating gastric content to measure its volume before feeding, followed by the injection of feeding via NEFT, contributes to the bidirectional circulation of microorganisms in the gut-NEFT axis (14–16). This microbial exchange is facilitated in preterm neonates due to two main factors that weakens their gastric barrier: (a) their gastric pH is neutral at birth (∼7) because of fetal ingestion of alkaline amniotic fluid (17); and (b) they exhibit longer gastric emptying times compared with full-term neonates (18). In fact, most of the nosocomial pathogens involved in NICU's outbreaks, including some species of the genera *Klebsiella, Serratia* or *Enterobacter* (19–21), are frequently found as NEFT-colonizers (7 17–21). A high abundance of these and other related genera usually precedes the onset of necrotizing enterocolitis (NEC), an inflammatory disease that poses a high mortality and morbidity burden in this population (22).

Since the gut microbiota is the factor with the greatest impact on the programming and maturation of the immune system in early life (5, 10), any disturbance in its development may condition their health in the short, medium, and long term. Therefore, the aim of this study is to evaluate the impact of NEFT-associated microbial communities in the establishment of preterm gut microbiota. For this, the initial colonization of the infant gut and NEFTs and its evolution during the first two weeks was studied using a 16S rRNA-based metataxonomic approach. The potential sharing of strains isolated from the same set of samples of bacterial species involved in NICU's outbreaks was assessed using a RAPD genotyping procedure.

## 2 Material and methods

### 2.1 Ethic statement

This study was approved by the Ethical Committee on Clinical Research of La Paz University Hospital, Madrid (Spain) on June 27th, 2018 (Code: HULP PI-3199). Collection of samples and clinical information was performed after obtaining informed written consent from the parents or legal representatives of the infants participating in this study.

### 2.2 Study design

A total of 42 preterm infants were initially recruited at the Service of Neonatology of La Paz University Hospital, Madrid (Spain) from April to October 2018. To detect significant variations in NEFTs bacterial colonization and abundance that may be clinically relevant, we set a threshold of a 1.5 log_10_ cfu/cm^2^ increase in the attached bacterial population during this period. Initially, a sample size of 33 subjects was estimated, to ensure the reliability and meaningfulness of our results (G*Power version 3.1.9.2, Universität Kiel, Germany; Wilcoxon signed-rank test for matched pairs, *α *= 0.05, power = 0.95, and effect size = 0.6). Taking into account a 30% dropout rate, the final sample size was increased to 42 subjects. The subjects were born at a gestational age of <32 weeks and admitted to the NICU. Exclusion criteria included preterm infants with any malformations or any congenital metabolic diseases. Immediately after birth, a NEFT was inserted in each infant to provide enteral feeding by gravity eight times a day (every 3 h). On average, each NEFT is inserted for a period of 48 h. The first NEFT (NEFT-1) was removed after 2 days from birth, the second NEFT (NEFT-2) was removed after the first hospitalization week (at day 9) and the third NEFT (NEFT-3) was removed after the second hospitalization week (at day 17). In parallel, samples of meconium (Me) and feces obtained after 2 days (F1), 9 days (F2) and 17 days (F3) of life were also collected from each patient (Supplementary Figure S1).

### 2.3 Sample collection, storage, and processing

All samples were collected by the medical and nursing staff of the Neonatology Unit. The fecal samples were collected from the diaper of each infant, using disposable sterile spatulas and sterile single-use gloves, placed in a sterile tube, and stored at −80 °C. NEFTs were removed from the infants, placed into individual sterile plastic bags and frozen at −80 °C. The collected samples were then transferred to the laboratory in containers provided of dry ice, and stored at −80 °C until processing.

### 2.4 DNA extraction from fecal samples

DNA extraction from feces was performed using a Maxwell® RSC instrument and a Maxwell® RSC PureFood GMO and Authentication Kit (Promega Corporation, Madison, WI, USA) as described by the manufacturer with the following modifications: 0.1 g of feces were placed into a 2 ml microcentrifuge tube and mixed with 0.8 ml of CTAB buffer (Promega Corporation, Madison, WI, USA) by vortexing for 30 s. Samples were heated at 95 °C for 15 min and allowed to cool for 5 min on the bench top. The content was transferred to a MP Biomedicals Lysing Matrix-A tube (MP Biomedicals, Santa Ana, CA, USA) and mechanically lysed using the MP Biomedicals FastPrep-24 Tissue and Cell Homogenizer (MP Biomedicals, Santa Ana, CA, USA) at a vertical velocity of 6 m/s for 3 cycles of 60 s. The lysate solution was treated with 32 μl of proteinase K (100 μg/ml) and 16 μl of RNase A (4 mg/ml) for 10 min at 70 °C. After incubation, the mixture was centrifuged at 16,000 × *g* for 5 min, and 300 μl of the supernatant were transferred to the Maxwell RSC Cartridge. The Maxwell RSC Pure Food Protocol was run following the indications of the manufacturer and the DNA was eluted from the samples in 100 μl of the elution buffer. DNA samples were frozen at -20 °C until the metataxonomic analysis was performed.

### 2.5 DNA extraction from the NEFTs

The internal content of a NEFT was obtained following the next protocol. Collected NEFTs were externally cleaned using 70% (v/v) ethanol. The residual liquid inside the tubes was firstly flushed into a 50 ml sterile tube by pumping air using a syringe. Then, 4 ml of 0.85% (w/v) sterile saline solution were injected into the same tube to remove weakly attached microorganisms. Finally, to fully detach the biofilms developed in the inner surface of the NEFTs, those were cut into 2 cm pieces using sterilized scissors that were collected into a new Falcon test tube containing 10 ml of sterile saline and vortexed for 1 min at maximum speed. Cells were harvested from the resulting suspension by centrifugation at 31,231 × *g* for 10 min at 4 °C. Pellets were suspended into 1 ml of sterile saline and stored at −80 °C until use. For DNA extraction, 1 ml aliquots were centrifuged at 15,000 × *g* for 10 min at 4 °C and the pellet was suspended into 400 µl of TE50 buffer (10 mM Tris-HCl: 50 mM EDTA, pH 8.0). The samples were then transferred to Fast-Prep tubes and processed at a Fast-Prep® FP120 equipment (Thermo) for 3 min at maximum speed. Then, tubes were centrifuged at 15,000 × *g* for 1 min at 4 °C, and the supernatants were transferred to a 2 ml Eppendorf tube. A mix of enzymes was added (120 µl of lysozyme [10 mg/ml], 4 µl of mutanolysine [10 KU/ml] and 3 µl of lysostaphin [4,000 KU/ml]) and blended with the samples. The samples were incubated at 37 °C for 90 min in a thermoblock. After that, 25 µl of Proteinase K (100 μg/ml) and 500 µl of AL Buffer (QIAamp® DNA Mini Kit 250) were added to the samples and incubated again at 56 °C for 30 min. Finally, 100 µl of 3M sodium acetate and 500 µl of 96% ethanol were added to each sample and mixed. DNA purification was performed using a NucleoSpin® Gel and the PCR Clean-up (QIAGEN) protocol. DNA was eluted in 25 µl of molecular grade water (Sigma). DNA samples were frozen at −20 °C until the metataxonomic analysis was performed.

### 2.6 Metataxonomic analysis of bacterial microbiota

A dual-barcoded 2-step PCR was used to amplify a fragment of the V3-V4 hypervariable region of the bacterial 16S ribosomal RNA gene. Barcodes were attached to 3′and 5′ends of the amplicons to allow separation between forward and reverse sequences. The DNA concentrations of the PCR products were pooled as equimolar and run on an agarose gel. The bands were excised and purified using the commercial QIAEX II Gel Extraction Kit (QIAGEN) and quantified with PicoGreen (BMG Labtech Jena, Germany). The sequencing of the aliquots of the purified barcoded DNA amplicons was performed using the Illumina MiSeq paired-end protocol (Illumina Inc., San Diego, CA) at the facilities of the Scientific Park of Madrid (Spain). Illumina software (version 2.6.2.3) was used to demultiplex the sequences according to the manufacturer's guidelines and pipelines. Bioinformatic performance and analyses were carried out combining QIIME 2 (version 2021.10) (23) and R software (version 3.5.1, https://www.r-project.org/). For denoising, the DADA2 pipeline (24) was used following this set: the forward reads were truncated at position 294 and their first 15 nucleotides were trimmed, while the reverse reads were truncated at position 276 and their first 10 nucleotides were trimmed, to discard positions for which nucleotide median quality was Q19 or below. Taxonomy data was assigned to Amplicon Sequence Variants (ASVs) using the q2-feature-classifier (25) employing classify-sklearn naïve Bayes taxonomy classifier with the SILVA 138.1 as reference database (26). To identify, visualize and remove contaminating DNA, the decontam package version 1.2.1 (27) was applied with a negative control sample. A table of ASVs, genera and phyla sequences per sample was generated, and bacterial taxa abundances were normalized with the Total Sum Scaling (TSS) normalization (relative abundance). After this normalization step, an additional filtering process was implemented where sequences showing a relative abundance of less than 0.01% were excluded from further analysis. This threshold was strategically chosen to focus on the more significant bacterial taxa and to ensure that the study was not impacted by the presence of extremely rare sequences.

### 2.7 RAPD-PCR genotyping of *Klebsiella*, *Serratia* and *Enterobacter* isolates

For isolates identification, 1 ml aliquot obtained after washing the internal contents of the probes was used. Decimal dilutions were made and 20 μl of each were plated in MacConkey (MCK, Oxoid) for isolation of *Enterobacteriaceae*. All plates were incubated 24 h at 37 °C under aerobic conditions. At least one representative colony of each morphology was selected and cultured on Brain Heart Infusion (Oxoid) agar for checking purity. Then, selected isolates were identified by Sanger sequencing of the 16S ribosomal RNA (rRNA). Purified PCR products were sequenced (STAB VIDA, Caparica, Portugal) and identified using BlastN (version 2.10) with the 16S rRNA database of NCBI.

All those isolates that were identified as belonging to species of the genera *Klebsiella*, *Serratia* and *Enterobacter* were submitted to genotyping profiling by Random Amplification of Polymorphic DNA [RAPD] procedure. A sample of 5 µl of genomic DNA of each isolate was used in sub-sequent PCR amplifications performed in an iCycler® thermocycler (Bio-Rad, California, USA). PCR conditions are shown in Supplementary Table S1. The primer used for the reaction was OPL5 (5′-ACGCAGGCAC-3′) (Sigma-Aldrich, Schnelldorf, Germany). Biomix Red® (Bioline Meridian, Memphis, USA) was used to perform the reaction. DNA band patterns were obtained after gel electrophoresis [2% (w/v) agarose gel] of the purified RAPD-PCR products (10 µl) obtained using the NucleoSpin® kit Gel and PCR Clean-up (Macherey-Nagel, Düren, Germany). Gels were run using a PowerPac Basic (Bio-Rad) and a Wide mini-sub cell GT (Bio-Rad) for 90 min at 45 V. Gel Red® Nucleic Acid Stain (Biotium, California, USA) (1:100) was used to visualize the DNA bands and the gels were observed in a Bio-Rad Universal Hood II transilluminator (Bio-Rad). Gel images were acquired using Gel Doc 1,000 Documentation System software (Bio-Rad). The band assignment of RAPD profiles was performed using Phoretix 1D Advanced software (Nonlinear Dynamics; Biostep, Jahnsdorf, Germany). Similarity between samples was compared using Phoretix 1D Database (Nonlinear Dynamics; Biostep, Jahnsdorf, Germany). Dendrograms were plotted using the unweighted pair group method with arithmetic averages (UPGMA) (28). Profiles were considered similar when the score values were >0.8.

### 2.8 Statistical analysis

Quantitative data were expressed as the median and interquartile range (IQR). The Shannon diversity index (29) was used to assess alpha diversity, which accounts for the quantity and evenness of ASVs, via the R vegan package (version: 2.5.6) (30). The Kruskal–Wallis test was performed for determining statistical differences among three or more groups, with Bonferroni *p*-value adjustment for pair-wise comparisons and Wilcoxon rank test was performed for determining statistical differences among two groups; a *p*-value < 0.05 was considered statistically significant. Values were expressed as median and quartiles 1 and 3 [Q1-Q3]. Beta diversity was studied using principal coordinates analysis (PCoA) for visually demonstrating patterns of bacterial profiles using a distance matrix filled with dissimilarity values. The Bray–Curtis and binary Jaccard indices were employed for quantitative and qualitative analyses, respectively. Permutational multivariate MANOVA (PERMANOVA) was used with 999 permutations. Heatmaps' cladograms were generated with Hclust hierarchical cluster analysis using the complete linkage method from the R's core “stats” package and the “ggplot2” package (31). Fisher's exact test was employed to evaluate correlations between NEFT and fecal metataxonomic data at a same sampling time. Kendall rank correlation coefficient was used to assess correlations between different genera within a same sample time (either NEFT or fecal samples).

## 3 Results

**Figure 1:**
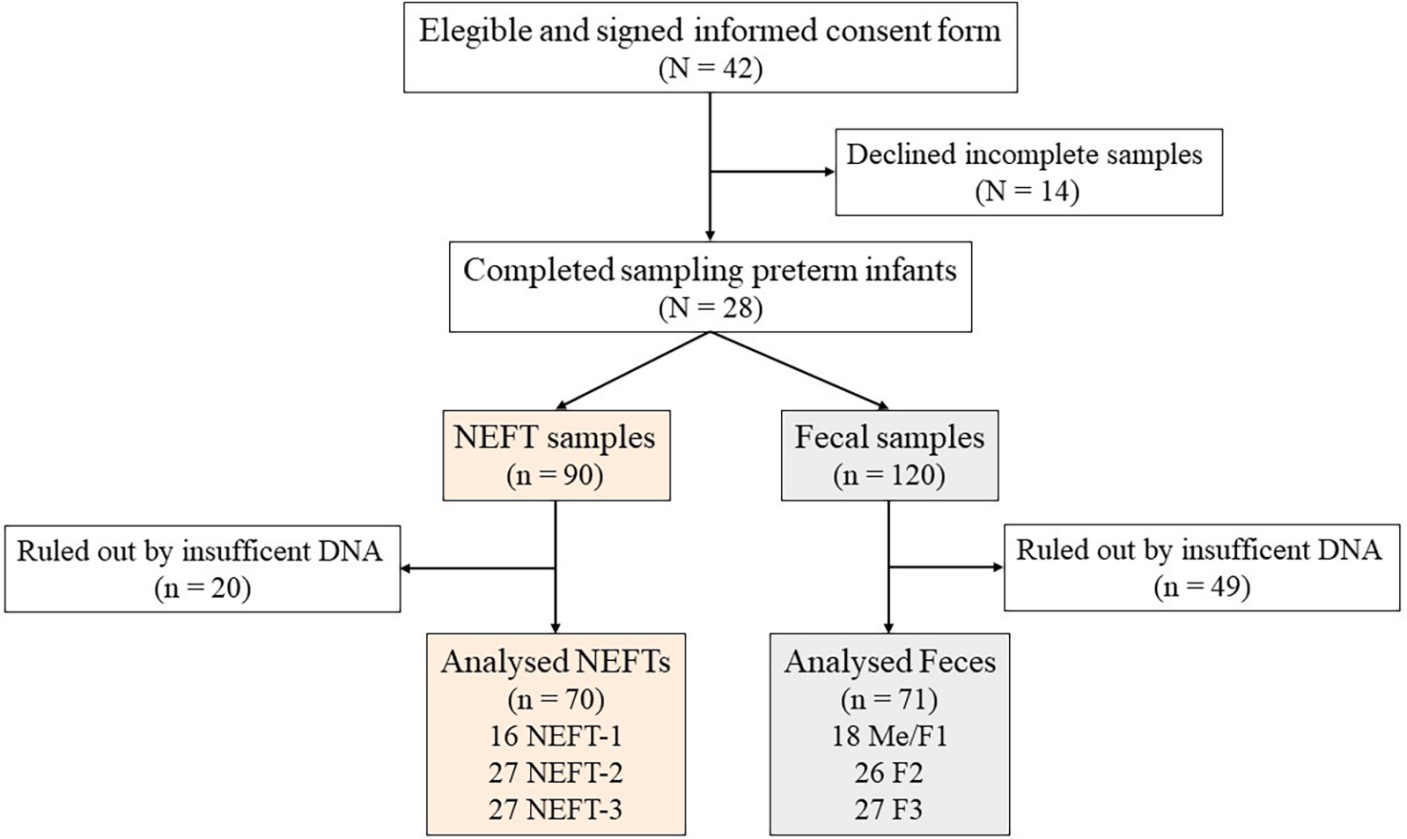
Study flowchart. Preterm infants included at the beginning and at the end of the study (**N**). Total collected nasogastric enteral feeding tube (NEFT) and fecal (**F**) samples, discarded samples and total number of samples finally analyzed (n).

The complete set of NEFT and fecal samples was only obtained from 28 subjects out of the 42 preterm infants initially recruited in this study (Figure 1). Clinical complications were the main reason for dropouts. The demographic and clinical characteristics of these 28 infants are summarized in Table 1. A total of 210 samples (90 NEFT-related samples and 120 fecal samples) were processed for DNA extraction. The first PCR round of the metataxonomic analysis revealed that only 141 samples (70 from NEFTs and 71 from feces) produced a single distinct electrophoretic band (Figure 1). Therefore, only amplicons from these 141 samples were submitted to the second PCR round and sequencing.

**Table 1:** Preterm clinical data. Preterm infant's clinical characteristics and demographic data (*N* = 28).

		N (%)
Gender	Male	9 (32)
	Female	19 (68)
Delivery type	Vaginal	6 (21)
	Cesarean	22 (79)
Pregnancy type	Single	11 (39)
	Multiple	17 (61)
Antibiotherapy	Yes	18 (64)
	No	10 (36)
Type of feeding	OMM	13 (46)
	DHM	5 (18)
	Mixed feed	10 (36)
Gestational age (weeks days)	Median	Min-Max
	30 ^3^	24^6^–31^6^
Birth weight (g)	Mean	Min-Max
	1.277	560–1,860

OMM, own mother's milk; DHM, donor human milk.

### 3.1 Metataxonomic analysis of the NEFT-related samples and evolution over time

The 16S rRNA-based metataxonomic analysis of the NEFT samples yielded 3,476,951 high-quality filtered sequences and the number of sequences per sample ranged between 13,908 and 83,114, with a median value of 50,432 sequences per sample. Overall, a total of 1,164 ASVs were identified.

**Figure 2:**
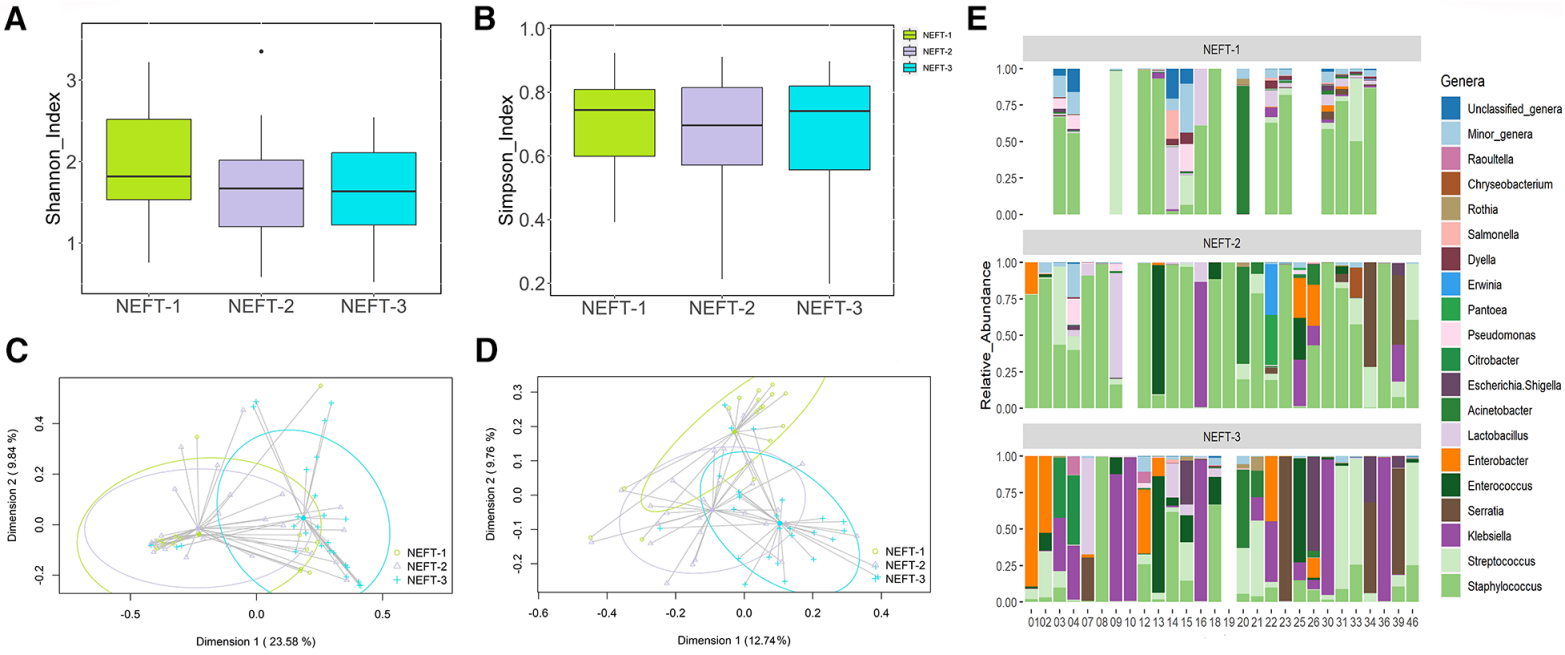
Comparative analysis of microbial diversity and composition of NEFTs samples over time. (**A**) Shannon Diversity Index (Kruskal-Wallis, *p* = 0.19). (**B**) Simpson Index (*p* = 0.89). (**C**) Two-dimensional principal coordinates analysis (2D-PCoA) of Bray-Curtis distances showing significant variance (PERMANOVA, *p* = 0.003). (**D**) Binary Jaccard-based 2D-PCoA also indicating significant differences (PERMANOVA, *p* = 0.003). (**E**) Relative abundance of the 18 dominant genera in preterm infants considered at the end of the study (*N* = 28) over time. This panel shows the distinct distribution and prevalence of each genus within each patient at different sampling times. Blank spaces in the bar plot indicate samples wherein insufficient genetic material was obtained (low DNA yield).

When analyzing alpha diversity over time, no statistical differences were detected among NEFT-1 [1.71 (1.38–2.14)], NEFT-2 [1.64 (1.20–2.98)], and NEFT-3 [1.59 (1.18-2.04)] samples (*p* = 0.56 and *p* = 0.95 for Shannon and Simpson indexes, respectively) (Figures 2A,B). However, significant differences were observed in relation to beta diversity; on the one hand, the Bray-Curtis distance matrix, which considers the relative abundance of ASVs, showed that NEFT-3 samples were significantly different when compared to those obtained at previous sampling times (*p* = 0.003). On the other hand, binary Jaccard distance matrix analysis, which considers the presence or absence of ASVs, also revealed significant differences among the different groups of NEFT samples according to the sampling time (*p* = 0.003) (Figures 2C,D).

Sequences belonging to 71 bacterial genera were detected in the NEFT samples. Among them, the 18 most abundant genera are shown in Figure 2E. NEFT-1 samples were dominated by sequences belonging to the genus *Staphylococcus*, representing 62.44% (39.79-84.54) of the total bacteria detected at this sampling point, followed by *Lactobacillus* [0.98% (0.28–3.43)]*, Streptococcus* [0.47% (0.12–4.53)], and *Acinetobacter* [0.43% (0.16–1.10)]. The relative abundances of these genera in NEFT-2 samples were similar to those found in the NEFT-1 ones. However, the abundance of *Staphylococcus* sequences was significantly lower (*p* < 0.05) in NEFT-3 samples [5.52% (0.59–14.84)], while that of some genera belonging to the Family *Enterobacteriaceae*, including *Klebsiella, Enterobacter*, and *Serratia*, increased in comparison to the previous samples. In addition, the relative abundance of minor genera in this type of samples gradually decreased over time.

### 3.2 Metataxonomic analysis of the fecal samples and evolution over time

**Figure 3:**
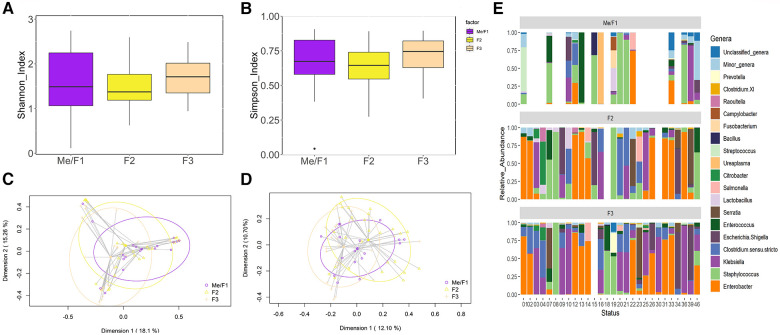
Comparative analysis of microbial diversity and composition of fecal samples (**F**) over time. (**A**) Shannon Diversity Index (Kruskal-Wallis, *p* = 0.39). (**B**) Simpson Index (*p* = 0.23). (**C**) Two-dimensional principal coordinates analysis (2D-PCoA) of Bray-Curtis distances showing significant variance (PERMANOVA, *p* = 0.001). (**D**) Binary Jaccard-based 2D- PCoA also indicating significant differences (PERMANOVA, *p* = 0.001). (**E**) Relative abundance of the 18 dominant genera in preterm infants considered at the end of the study (*N* = 28) over time. This panel shows the distinct distribution and prevalence of each genus within each patient at different sampling times. Blank spaces in the bar plot indicate samples wherein insufficient genetic material was obtained Blank samples indicate instances where insufficient genetic material was obtained (low DNA yield).

The metataxonomic analysis of the fecal samples yielded 1,683,527 high-quality filtered sequences, ranging from 4,956 to 38,090 sequences per sample (median value: 22,278 sequences per sample). In this case, a total of 173 ASVs were identified. Alpha diversity analysis did not reveal any statistical difference among Me/F1 [1.37 (1.05–2.06)], F2 [1.34 (1.16–1.74)], and F3 [1.70 (1.35–2.01)] samples (*p* = 0.18 and *p* = 0.12 for Shannon and Simpson indexes, respectively), indicating a similar level of species diversity within these groups (Figures 3A,B). However, the Bray-Curtis distance matrix found significant differences between Me/F1 and F3 samples (*p* = 0.017). While the binary Jaccard distance matrix showed differences in F3 when compared to Me/F1 and F2 samples (*p* = 0.003 and *p* = 0.018, respectively). Me/F1 samples also differed statistically with F2 samples (*p* = 0.003) (Figures 3C,D).

Sequences corresponding to 47 different genera were detected in the fecal samples. Among them, the 18 most abundant bacterial genera in the samples of the 28 patients are represented in Figure 3E. Me/F1 samples were characterized by the presence of sequences of the genera *Staphylococcus* [14.44% (2.07–50.77)] and, in comparison to other sampling times, by a higher abundance of the minor genera group [0.74% (0.08-8.54)]. In F2 samples, the relative abundance of *Staphylococcus* spp. decreased up to 5.98% [1.54–21.20]. This downward trend was later confirmed in F3 samples in which *Staphylococcus* relative abundance significantly (*p* < 0.05) drops to 0.45% [0.06–3.29]. Parallel, an increase in the relative abundance of different genera of *Enterobacteriaceae* (*Enterobacter*, *Klebsiella* or *Escherichia/Shigella*) was observed in F3 samples (Figure 3E). Similarly, *Clostridium sensu stricto* relative abundance tended to increase over time from 0.45% [<0.01–2.66] in Me/F1 to 6.92% [<0.01–20.02] in F3 samples, although no significant differences were observed (*p* = 0.10).

### 3.3 Comparison of NEFT and fecal metataxonomic data

The assessment of potential correlations between the NEFT and fecal sequences obtained in the same sampling time was carried out excluding NEFT-1 and Me/F1 samples because the DNA concentration in these was extremely low. Such assessment was focused on the most frequent and/or abundant genera found in this study (*Staphylococcus, Streptococcus, Klebsiella, Serratia, Enterococcus,* and *Lactobacillus*).

**Figure 4:**
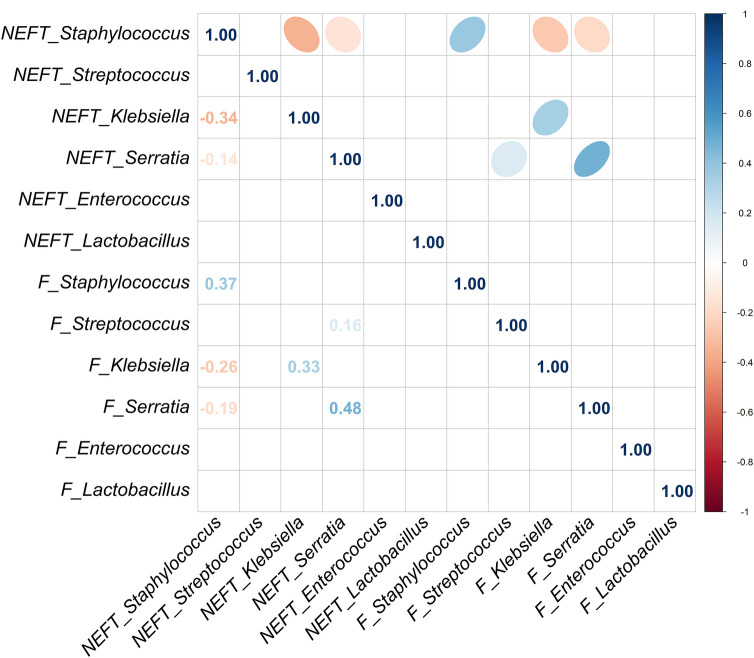
Correlation analysis between NEFT and feces samples taken at day 9 of life (NEFT-2 and F2). Correlation matrix of six relevant bacterial genera (*Staphylococcus*, *Streptococcus*, *Klebsiella*, *Serratia*, *Enterococcus* and *Lactobacillus*) at sampling time 2. This diagram shows Kendall rank correlations in the upper right panel and their corresponding correlation coefficients in the lower left panel. Only significant (*p* < 0.05) correlations are included.

Strong significant associations were observed for the presence of both *Staphylococcus* (*p* = 0.001) and *Enterococcus* (*p* = 0.002) in NEFT-2 and F2 (Table 2), whilst no significant associations were identified for *Streptococcus*, *Klebsiella*, and *Lactobacillus* (*p* > 0.05). A non-significant trend for association between these two types of samples was found for *Serratia* (*p* = 0.081). When comparing NEFT-2 and F2 samples for potential interactions, significant positive correlations were observed between the relative abundances of *Serratia* (r = 0.48, *p* < 0.05), *Staphylococcus* (r = 0.37, *p* < 0.05) and *Klebsiella* (r = 0.33, *p* < 0.05) (Figure 4). Subsequently, both types of samples were analyzed independently (either NEFTs or feces) (Figure 4). In NEFT-2 samples, a significant negative correlation was found between the relative abundances of *Staphylococcus* and *Klebsiella* (r = −0.34, *p* < 0.05), and between those of *Staphylococcus* and *Serratia* (r = −0.14, *p* < 0.05). In contrast, no significant correlations were observed between these pairs of genera in F2 samples (Figure 4).

**Table 2:** Presence/absence analysis in samples. Simultaneous presence/absence analysis in NEFT and fecal samples.

Bacterial genera		Feces							
		Sampling time 2 (9 days)				Sampling time 3 (17 days)			
		Presence	Yes	No	*p*-value*	Presence	Yes	No	*p*-value*
NEFT	*Staphylococcus* spp.	Yes	25	0	0.001	Yes	20	6	0.001
		No	0	0		No	0	0	
	*Streptococcus* spp.	Yes	8	13	0.188	Yes	17	8	0.346
		No	0	4		No	0	1	
	*Klebsiella* spp.	Yes	6	8	0.190	Yes	17	5	0.072
		No	2	9		No	1	3	
	*Serratia* spp.	Yes	3	5	0.081	Yes	7	6	0.015
		No	1	16		No	1	12	
	*Enterococcus* spp.	Yes	9	0	0.002	Yes	13	1	0.117
		No	6	10		No	8	4	
	*Lactobacillus* spp*.*	Yes	7	13	0.161	Yes	7	10	0.128
		No	0	5		No	1	8	

Detection of six relevant bacterial genera (*Staphylococcus*, *Streptococcus*, *Klebsiella*, *Serratia*, *Enterococcus*, and *Lactobacillus*) in feces samples taken at day 9 (NEFT-2/F2) and day 17 of life (NEFT-3/F3).

* Significant correlations of the presence of specific bacterial genera in both locations were considered at *p* < 0.05 (Fisher's exact test).

**Figure 5:**
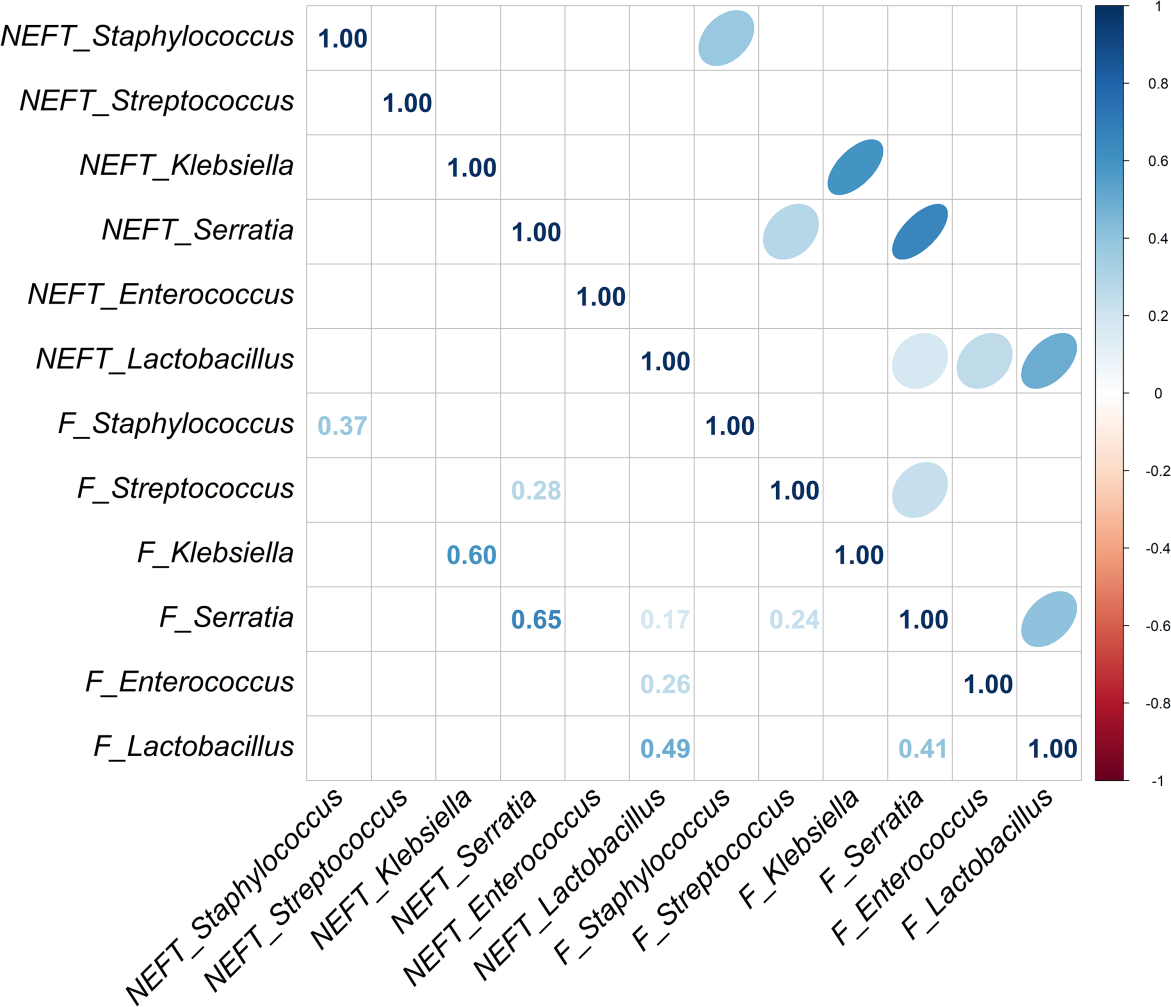
Correlation analysis between NEFT and feces samples taken at day 17 of life (NEFT-3 and F3). Correlation matrix of six relevant bacterial genera (*Staphylococcus*, *Streptococcus*, *Klebsiella*, *Serratia*, *Enterococcus* and *Lactobacillus*) at sampling time 3. This diagram shows Kendall rank correlations in the upper right panel and their corresponding correlation coefficients in the lower left panel. Only significant (*p* < 0.05) correlations are included.

A strong significant association was also observed for the presence of *Staphylococcus* (*p* = 0.001) in NEFT-3 and F3 samples, while the association regarding *Serratia* sequences reached a statistical significance, too (*p* = 0.015) (Table 2). No significant associations were found for *Enterococcus* (*p* = 0.117), *Lactobacillus* (*p* = 0.128) and *Streptococcus* (*p* = 0.346). In this case, a non-significant trend for association between these two types of samples was found for *Klebsiella* (*p* = 0.072). A comparison of NEFT-3 and F3 samples for potential interactions, revealed a strong correlation in the relative abundances of *Serratia* in both sites (r = 0.65, *p* < 0.05). Positive correlations were also observed between the relative abundances of *Klebsiella* (r = 0.60, *p* < 0.05), *Staphylococcus* (r = 0.37, *p* < 0.05), and *Lactobacillus* (r = 0.49, *p* < 0.05), in both types of samples (Figure 5). Regarding NEFT-3 samples, no significant correlations were observed among the different genera detected in the same sample type. In contrast, significant positive correlations were found between the relative abundances of *Lactobacillus* and *Serratia* (r = 0.41, *p* < 0.05) and, also, between those of *Streptococcus* and *Serratia* (r = 0.24, *p* < 0.05) (Figure 5).

### 3.4 RAPD genotyping of NEFT and fecal isolates belonging to species of the genera *Klebsiella*, *Serratia* and *Enterobacter*

To investigate if bacterial transference from NEFT to feces and/or vice versa might have occurred, the isolates belonging to the genera *Klebsiella*, *Serratia* and *Enterobacter* were submitted to RAPD genotyping. The results are shown in Tables 3, 4 and in Supplementary Figure S2. Globally, 7 patients (28%) shared at least one RAPD-PCR profile in fecal and NEFT samples. The 11% of the profiles were found at least once simultaneously in NEFT and fecal samples from the same patient.

**Table 3:** *Klebsiella* spp. RAPD-PCR genetic profiles found in the collected samples over time.

	Profile	No. of isolates	Detected in NEFT	Detected in feces	Factor		
					Same patient	Time persistance	Box shared
*Klebsiella pneumoniae*	1	1	–	1	Yes	No	Yes
	2	11	–	11	No	Yes	No
	3	3	3	–	No	No	Yes
	4	7	7	–	No	Yes	No
	5	4	–	4	No	Yes	Yes
	6	5	–	5	No	No	No
	7	1	–	1	No	No	Yes
	8	1	–	1	No	No	Yes
	9	4	–	4	No	No	No
*Klebsiella oxytoca*	1	1	1	–	Yes	No	Yes
	2	1	–	1	Yes	No	Yes
	3	2	–	2	Yes	Yes	Yes
	4	2	–	2	No	No	No
*Klebsiella michiganensis*	1	3	–	3	No	Yes	Yes
	2	3	3	–	No	No	No
	3	4	–	4	No	Yes	No
	4	2	–	2	No	No	Yes
	5	2	–	2	Yes	No	Yes
	6	3	–	3	No	No	No
	7	1	1	–	Yes	No	Yes
	8	3	3	–	No	Yes	No
	9	1	–	1	Yes	No	Yes
	10	1	1	–	Yes	No	Yes
	11	1	–	1	Yes	No	Yes

RAPD-PCR genetic profile analysis of *Klebsiella pneumoniae* (9)*, K. oxytoca* (4) and *K. michiganensis* (11) isolates grouped by shared characteristics (patient, box) and time persistence. Nasogastric enteral feeding tube (NEFT) and fecal (F) samples.

**Table 4:** *Serratia* spp. and *Enterobacter hormaechei* RAPD-PCR genetic profiles found in the collected samples over time.

	Profile	No. of isolates	Detected in NEFT	Detected in feces	Factor		
					Same patient	Time persistance	Box shared
*Serratia nematodiphilia*	1	5	2	3	No	Yes	No
	2	3	1	2	No	No	Yes
	3	3	2	1	No	No	No
	4	1	1	–	Yes	No	Yes
	5	1	1	–	Yes	No	Yes
	6	3	2	1	No	No	No
	7	1	–	1	Yes	No	Yes
	8	7	2	5	No	No	No
*Serratia marcescens*	1	2	–	2	No	No	No
	2	4	3	1	No	No	No
	3	5	2	3	Yes	Yes	Yes
*Serratia liquifaciens*	1	1	1	–	Yes	No	Yes
	2	2	1	1	Yes	No	Yes
*Enterobacter hormaechei*	1	1	1	–	Yes	No	Yes
	2	1	–	1	Yes	No	Yes
	3	17	5	12	No	Yes	No

RAPD-PCR genetic profile analysis of *Serratia nematodiphilia* (8), *S. marcescens* (3), *S. liquifaciens* (2) and *Enterobacter hormaechei* (3) isolates grouped by shared characteristics (patient, box) and time persistence. Nasogastric enteral feeding tube (NEFT) and fecal (F) samples.

In the case of *Klebsiella* isolates (*n* = 67) RAPD-PCR revealed the existence of 9, 4 and 11 different RAPD profiles for *Klebsiella pneumoniae* (37 isolates), *Klebsiella oxytoca* (6 isolates) and *Klebsiella michiganensis* (24 isolates), respectively (Table 3 and Supplementary Table S1). Overall, 9 out of the 24 RAPD profiles (37.5%) were ascribed to a single isolate. RAPD profiles 2, 4 and 6 of *K. pneumoniae* were shared by 11 (∼ 30%), 7 (∼ 20%) and 5 (∼14%) isolates, respectively. Isolates ascribed to profiles 2 and 6 of *K. pneumoniae* were recovered from fecal samples of different patients who did not share the same hospitalization box. A similar pattern was observed for profile 9 of *K. pneumoniae*, profile 4 of *K. oxytoca* and profiles 1, 3, 6 of *K. michiganensis*. Similarly, some of the *Klebsiella* profiles were shared by isolates recovered from NEFTs of patients who were hospitalized in different boxes (profile 4 of *K. pneumoniae* and profile 2 of *K. michiganensis*)*.* Profiles 3 and 5 of *K. pneumoniae* were found in isolates recovered from NEFT and feces samples, belonging to patients sharing the same box. This pattern was also observed for profiles 1 and 4 of *K. michiganensis.* Overall, 15 out of the 24 *Klebsiella* profiles (62.5%) were detected at the same sampling time in fecal samples of different patients, and 7 out of 24 (29.2%) persisted over time in the same patient.

The *Serratia* isolates were identified as *Serratia nematodiphilia* (24 isolates), *Serratia marcescens* (11 isolates) and *Serratia liquifaciens* (3 isolates). These isolates were classified in 8, 3 and 2 different RAPD profiles, respectively (Table 4 and Supplementary Figure S2). Overall, 4 of the 13 genotypes corresponded to a single isolate. Profile 3 of *S. marcescens* and profile 2 of *S. liquifaciens* were detected simultaneously in NEFT and fecal samples of the same patient while profiles 1, 2, 3, and 8 of *S. nematodiphilia* and profile 2 of *S. marcescens* were detected simultaneously in NEFT and fecal samples of different patients. Seven RAPD profiles (53.8%) were found in samples from different patients at the same sampling time, although only 2 (15.4%) of the *Serratia* profiles persisted in the same patient over time. Overall, 46.2% of the *Serratia* profiles were found in patients who did not share hospitalization boxes (Table 4).

The 19 isolates of the genus *Enterobacter* were identified as *Enterobacter hormaechei.* Only 3 different RAPD profiles were detected among these isolates and most of them were ascribed to profile 3 (89.4%) (Table 4 and Supplementary Figure S2). *Enterobacter* isolates sharing this profile were recovered from both NEFT and fecal samples and it was simultaneously detected in almost the totality of patients (5 of 6) either sharing or not the same hospitalization box. In some of these patients (2), this profile was detected over time both in NEFT-2 and NEFT-3, and F2 and F3.

## 4 Discussion

Staying at a NICU for some weeks after birth, is critical for the survival of many preterm neonates. However, this prolonged stay often results in an impairment of their gut colonization process because of the idiosyncratic environment in which they reside and the management practices that are typically implemented in such facilities (1, 16, 32). The exposure to high risk nosocomial pathobionts, together with an alteration of the gut microbiota and an immature immune system, predispose these infants to inflammation, sepsis and NEC, and may have a short-, medium- and long-term negative impact on the host's health (33). To better understand the actual impact of NICU's staying in the early colonization of the gut and NEFTs by potential nosocomial bacteria, a metataxonomic analysis of fecal and NEFT-related samples obtained during the first 2 weeks of life of preterm infants was performed.

NEFT-related samples obtained at each sampling point (NEFT-1, NEFT-2 or NEFT-3) exhibited a similar degree of bacterial diversity in their bacterial communities. However, differences in beta diversity highlighted relevant shifts in the composition and structure of the NEFT bacteriome over time, especially when the NEFT-3 metataxonomic data were compared with those of the rest of the samples. While NEFT-1 and NEFT-2 samples were characterized by a very high dominance of sequences belonging to *Staphylococcus* and, to a lower extent, to other Gram-positive genera, the relative abundance of *Staphylococcus* sequences was notably lower in NEFT-3 samples. Nevertheless, it is still the most abundant genus at the end of the study along with Gram-negative genera, such as *Klebsiella*, *Serratia* or *Enterobacter*. Previous works have reported that the genera cited above are commonly found in the inner surfaces of NEFTs and/or in preterm feedings passed through NEFTs (34–41).

The composition of the fecal bacterial communities also changed during the first two weeks of life of the recruited preterm infants. Presence and abundance of *Staphylococcus* sequences was a feature of the meconium and fecal samples analyzed in this work, increasing from Me/F1 samples but decreasing from F2 to F3 samples. In contrast, the abundance of sequences corresponding to some genera of the family *Enterobacteriaceae* (*Enterobacter*, *Klebsiella* or *Escherichia/Shigella*) increased over time and were highest in F3 samples. Sequences of *Enterococcus* and *Streptococcus* were also frequently detected among the analyzed fecal samples. Overall, al the genera cited above have been described as common members of the preterm gut microbiota (32, 41, 42). Both the predominance of coagulase-negative staphylococci in feces obtained in the first days of life and their subsequent decrease, concomitant with an increase in gut enterobacteria have already been described in preterm and term infants, independently of their type of delivery or feeding (43–47).

Sequences belonging to the genera *Lactobacillus* (including the different species in which this genus was reclassified in 2020 (48) and *Bifidobacterium* were scarcely detected in the fecal samples of the recruited infants, in contrast to what is described in full-term breastfed infants (49, 50). Bifidobacteria and lactobacilli can successfully compete with nosocomial pathogens in the gut microbiota, including those belonging to some genera of the family *Enterobacteriaceae*, such as *Klebsiella* (51, 52)*.* It is important to note that the presence of sequences of the genus *Clostridium* in the fecal samples of preterm infants, but not in those collected from NEFTs, is consistent with differences in oxygen concentration between both environments.

The results of our work suggest that staphylococci, which are widespread in the hospital environment and in the skin and mucosal surfaces of the hospital staff (53–56), may play a pioneering role in the NEFT colonization process. The main species found in these settings (*Staphylococcus epidermidis* and other coagulase-negative species, *Staphylococcus aureus*) can form biofilms within medical devices, including NEFTs. Within a few hours, they could create a basic biofilm structure that may facilitate the adhesion and colonization of other nosocomial bacteria, such as the enterobacterial genera studied in this work. However, studies dealing with interactions between staphylococci and enterobacteria in real hospital devices are lacking.

Within the first few days after birth, there is an intensification in the gut colonization process (57). Having in mind the NICUs routine practices for preterm feeding (see Introduction), it is not strange that the bacterial community associated to the inner surface of NEFTs becomes closer to that found in the infant feces as the hospitalization length increases. Each NEFT of this study remains in place for an average of 48 h, time more than enough for the formation of a complex multispecies biofilm inside these devices (35–37). The simultaneous presence of relevant nosocomial pathogens in the biofilms formed inside NEFTs within two weeks after birth, poses a threat on the host health (21), since they include the main agents involved in neonatal sepsis (56, 58–62). In addition, microbes living in complex multispecies biofilms are usually more resistant to antimicrobials and other adverse circumstances because of different mechanisms (63–68), including an enhanced horizontal transfer of antibiotic resistance genes (69–73).

Although intravascular catheters are considered a major source of bacteremia in preterm neonates, several cases of late-onset sepsis (LOS) remain without an identified source (74). In this frame, the results of our work, including the sharing of pathobionts' strains between preterm feces and NEFTs even in patients staying in different boxes, suggest that these devices should receive more attention as potential sources of bacteremia-causing microbes. In fact, NEFT-associated communities may be critical for the prevention or predisposition to NICU-related infections. Coagulase-negative staphylococci (mainly *S. epidermidis*) may play a pivotal role in such dichotomy. This species has emerged as the predominant LOS-causing pathogen in preterms (75, 76). On the other hand, it has important roles in early life, from inhibiting more virulent pathogens, including *S. aureus*, *K. pneumoniae*, *K. oxytoca* or *S. marcescens* (42–45), to educate the innate immune system (77). The different outcome may depend on the strain properties (biofilm formation ability, antimicrobial resistance, and other features) and the environment, since this species is equipped with a remarkable genetic flexibility that may allow a rapid adaptation to a changing environment (78, 79). A deeper characterization of these microbial communities at the strain level, will be though necessary to better predict their potential for preventing or causing problems in a NICU setting.

In the same context, some enterobacteria, including specific strains of *Klebsiella*, can produce antimicrobial agents that are particularly active against other strains of the same species (77, 80, 81), including multi-drug resistant strains responsible for preterm mortality (82). NEFTs may also be the source of lactic acid bacteria, which may arise from inoculating OMM through these devices, with potential to modulate the process of NEFT colonization. As an example, in a previous work we showed that *Ligilactobacillus salivarius* 20SNG3 was effective in modulating the ability of biofilm formation of NEFT-isolated *K. pneumoniae* and *S. marcescens* strains (83).

The main limitations of our preliminary study are a slightly higher dropout rate than expected (33% vs. 30%) and the low concentration of DNA detected in NEFTs and fecal samples collected 48 h after birth, which considerably reduced the number of samples analyzed. However, these initial pairwise samples (*i.e.,* NEFT-1 and Me/F1) are extremely valuable as this study demonstrated, for the first time, that microbial interactions occur between these two niches within such a short time span, during a critical period of life for preterm infants. Moreover, it should be stated that the number of isolates that share the same profile could have been underestimated as the criteria for selecting these is based on morphology and some strains belonging to the same species could have shared the same morphology.

## 5 Conclusions

NEFT-associated microbial communities exert a significant influence on the early bacterial colonization of the gut of preterm infants. Microbial profiles of certain strains of *Klebsiella* spp., *Serratia* spp. and *Enterobacter* spp., often implicated in NICU's outbreaks, were detected in both NEFTs and fecal samples from the same patient, suggesting a direct exchange of these pathogens between both environments. This could facilitate the establishment of an aberrant gut microbiota in these children.

Furthermore, our results have demonstrated that certain strains are shared among preterm infants hospitalized in different boxes, suggesting a potential exchange of high-risk species within the NICU. This study may assist clinical staff in implementing best practices to mitigate the spread of pathogens that could jeopardize the health of preterm infants.

## Data Availability

The original contributions presented in the study are included in the article/**Supplementary Material**, further inquiries can be directed to the corresponding author.
