# Supplementary material for: Linking preterm infant gut microbiota to nasograstric enteral feeding tubes: exploring potential interactions and microbial strain transmission
Source: Front Pediatr. 2024 Jun 17;12:1397398. doi: 10.3389/fped.2024.1397398 (PMC11215057; doi:10.3389/fped.2024.1397398)
Supplement: Supplementary file 1 [file Table1.pdf]

Supplementary Table 1: RAPD-PCR program: Settings for RAPD-PCR fingerprinting of microorganisms isolated from NEFTs and fecal samples over time.

Supplementary Table 1

**RAPD-PCR program settings**			
**Steps**		**Temperature (ºC)**	**Time**
**Denaturalization**		94	3 min
15 cycles	Denaturalization	94	30 s
	Annealing	30	30 s
	Extension	72	1 min
**Denaturalization**		94	4 min
25 cycles	Denaturalization	94	30 s
	Annealing	30	1 min
	Extension	72	1 min + 25 s
**Maintenance**		8	∞
