# Supplementary material for: Linking preterm infant gut microbiota to nasograstric enteral feeding tubes: exploring potential interactions and microbial strain transmission
Source: Front Pediatr. 2024 Jun 17;12:1397398. doi: 10.3389/fped.2024.1397398 (PMC11215057; doi:10.3389/fped.2024.1397398)
Supplement: Supplementary file 2 [file Image1.pdf]

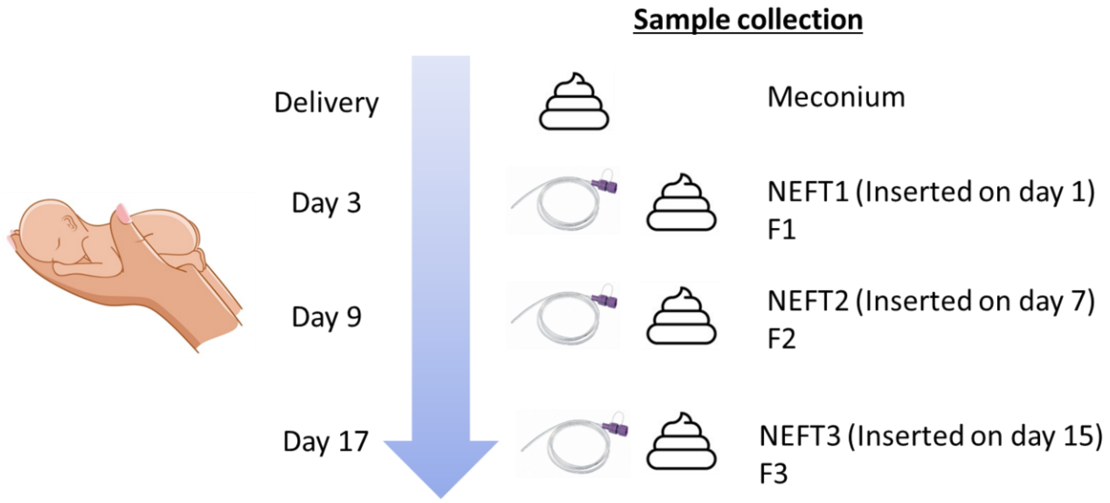

Supplementary Figure 1: Experimental design of samples collection. Meconium (Me), fecal (F) and nasogastric enteral feeding tube (NEFT) samples were collected over the first 17 days of preterm's life. NEFTs were inserted and stay inside the preterm infants on average 48 ± 1 h (mean value).
