# Supplementary material for: Linking preterm infant gut microbiota to nasograstric enteral feeding tubes: exploring potential interactions and microbial strain transmission
Source: Front Pediatr. 2024 Jun 17;12:1397398. doi: 10.3389/fped.2024.1397398 (PMC11215057; doi:10.3389/fped.2024.1397398)
Supplement: Supplementary file 3 [file Image2.pdf]

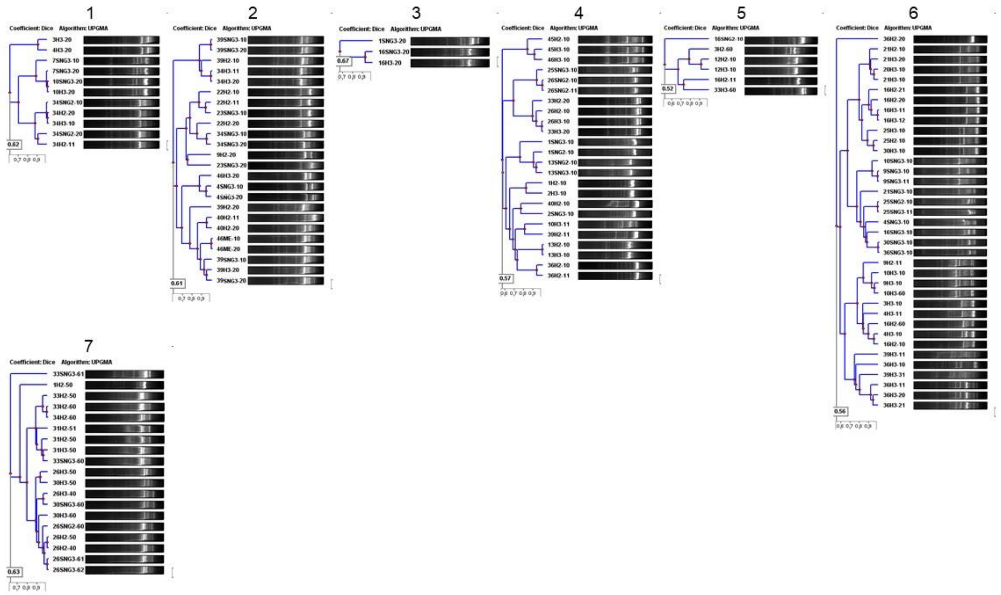

Supplementary Figure 2: **RAPD profiles analysis:** Dendrogram showing the genetic relationship among the *Serratia marcescens* (1), *S. nematodiphilia* (2), *S. liquifaciens* (3), *Klebsiella michiganensis (*4), *K. oxytoca* (5), *K. pneumoniae* (6) and *Enterobacter hormaechei* (7) isolates based on the Dice's coefficient (Algorithm UPGMA).
